# Fatal disease associated with Swine *Hepatitis E virus* and *Porcine circovirus 2* co-infection in four weaned pigs in China

**DOI:** 10.1186/s12917-015-0375-z

**Published:** 2015-03-26

**Authors:** Yifei Yang, Ruihan Shi, Ruiping She, Jingjing Mao, Yue Zhao, Fang Du, Can Liu, Jianchai Liu, Minheng Cheng, Rining Zhu, Wei Li, Xiaoyang Wang, Majid Hussain Soomro

**Affiliations:** Laboratory of Animal Pathology and Public Health, College of Veterinary Medicine, China Agricultural University; Key Laboratory of Zoonosis of Ministry of Agriculture, China Agricultural University, Beijing, 100193 China; Department of Veterinary Medicine, Laboratory of Animal Histology and Anatomy, College of Agriculture, Hebei University of Engineering, Handan, Hebei 056021 China

**Keywords:** Weaned pigs, *Hepatitis E virus*, *Porcine circovirus 2*, Co-infection, High mortality

## Abstract

**Background:**

In recent decades, *Porcine circovirus 2* (PCV2) infection has been recognized as the causative agent of postweaning multisystemic wasting syndrome, and has become a threat to the swine industry. *Hepatitis E virus* (HEV) is another high prevalent pathogen in swine in many regions of the world. PCV2 and HEV are both highly prevalent in pig farms in China.

**Case presentation:**

In this study, we characterized the HEV and PCV2 co-infection in 2–3 month-old piglets, based on pathogen identification and the pathological changes observed, in Hebei Province, China. The pathological changes were severe, and general hyperemia, hemorrhage, inflammatory cell infiltration, and necrosis were evident in the tissues of dead swine. PCR was used to identify the pathogen and we tested for eight viruses (HEV, *Porcine reproductive and respiratory syndrome virus*, PCV2, *Classical swine fever virus*, *Porcine epidemic diarrhea virus*, *Transmissible gastroenteritis coronavirus, Porcine parvovirus* and *Pseudorabies virus*) that are prevalent in Chinese pig farms. The livers, kidneys, spleens, and other organs of the necropsied swine were positive for HEV and/or PCV2. Immunohistochemical staining showed HEV- and PCV2-antigen-positive signals in the livers, kidneys, lungs, lymph nodes, and intestine.

**Conclusion:**

HEV and PCV2 co-infection in piglets was detected in four out of seven dead pigs from two pig farms in Hebei, China, producing severe pathological changes. The natural co-infection of HEV and PCV2 in pigs in China has rarely been reported. We speculate that co-infection with PCV2 and HEV may bring some negative effect on pig production and recommend that more attention should be paid to this phenomenon.

## Background

The rapid development of the pig industry in China accompanies with outbreaks of epidemic diseases in recent years. *Hepatitis E virus* (HEV) has been identified on pig farms in many regions of the world, including China [1-3]. HEV seropositivity rates of 76.6% and 90% have been reported in pig herds of large-scale and family-scale farms in China, respectively [4]. Increasing evidence indicates that HEV can infect both humans and animal [5]. To date, most studies of HEV based on prevalence surveys, and research into HEV-associated mortality during natural infection was limited. Mao et al. reported that co-infection with HEV and *Porcine reproductive and respiratory syndrome virus* (PRRSV) could lead to high mortality in swine [6], and they speculated that co-infection with HEV and other pathogens could cause serious disease. It has been demonstrated that HEV and *Porcine circovirus 2* (PCV2) could cause infectious hepatitis, but swine naturally co-infected with HEV and PCV2 in China has rarely been reported [3,7,8]. PCV2 infection occurs in many countries and poses a considerable threat to the swine industry [9]. Although the recently research showed that infection of PCV2 could be effectively reduced by utilizing PCV2 vaccine [10], prevention of PCV2 in the pig production should be paid more attention. In the present study, pathogen identification and the observation of pathological changes demonstrated a natural co-infection with HEV and PCV2 in the swine on two pig farms in Hebei Province, China. This discovery may provide a new perspective for clinical research.

## Case presentation

### Medical history and clinical symptoms

From November to December 2013, an outbreak of an unknown disease occurred at two small-scale pig farms (103 pigs in farm A and 101 pigs in farm B), operating for a short time in Hebei Province, China. All of the piglets fed in both pig farm A and B were aged 2–3 months. Pig farm A reported the deaths of 93 piglets (mortality rate was 90.3%), and pig farm B the deaths of 90 pigs (mortality rate was 89.1%). The affected animals on both farms presented with symptoms of fever, dyspnea, diarrhea, and anorexia. In pig farm A, the veterinary administrated timicosin and doxycycline to treat the pigs. And in pig farm B, florfenicol was administrated. However, the swine did not respond to antibiotic treatment.

### Sampling and pathological changes

Necropsies were performed on seven dead piglets: three from farm A (pigs 1, 2, and 3) and four from farm B (pigs 4, 5, 6, and 7). The tissues examined included the liver, spleen, lung, kidney, heart, intestine, and lymph nodes. All tissues used for histological examination were fixed in 2.5% (w/v) glutaraldehyde–polyoxymethylene solution for 48 h. The fixed tissues were routinely processed, embedded in paraffin, sectioned (4 μm thickness), and stained with hematoxylin and eosin. Portions of the liver, spleen, kidney, brain and lung tissues were used for pathogen detection and stored at −80°C until required.

### Gross lesions

**Figure 1:**
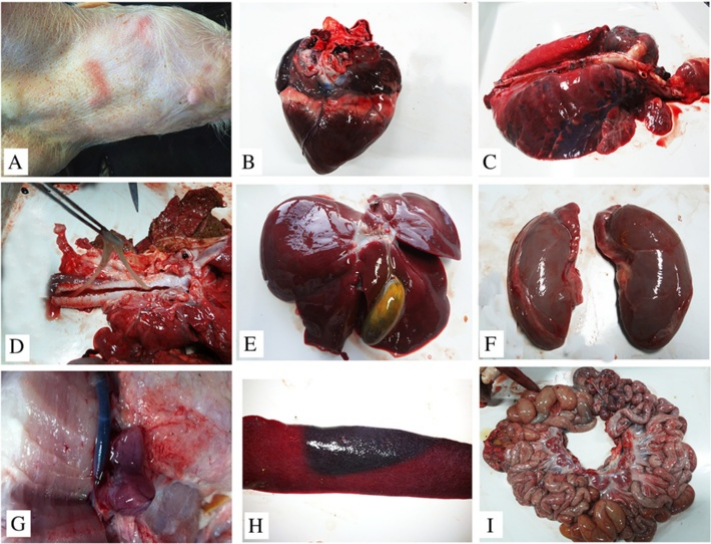
**Necropsy observations. (A)** Hemorrhage in the skin. **(B)** Dilated right ventricle of the heart. **(C, D)** Lung with hemorrhage, hyperemia, necrosis, and tracheal exudate. **(E)** Dark red and swollen liver. **(F)** Enlarged kidneys. **(G)** Clearly swollen lymph node. **(H)** Enlarged spleen, with infarction. **(I)** Enlarged mesenteric lymph nodes.

Seven dead piglets were necropsied and diagnosed. Scattered hemorrhagic spots were observed on the surface of the skin (Figure 1A). The right ventricle was dilated so that the ratio of the transverse/longitudinal diameters was increased (Figure 1B). Hyperemia, hemorrhage, and necrosis were present in large local areas of the lung (Figure 1C). A transparent gelatinous exudate was observed in the trachea (Figure 1D). The liver was enlarged and the surface was a dark red color (Figure 1E). It was difficult to strip the kidney capsule, and all the kidneys showed varying degrees of enlargement (Figure 1F). The lymph nodes and spleens were swollen to varying degrees (Figure 1G, H). Hemorrhage and infarction were observed in the spleen (Figure 1H). The mesenteric lymph nodes were enlarged and hyperemic (Figure 1I).

### Histological lesions

**Figure 2:**
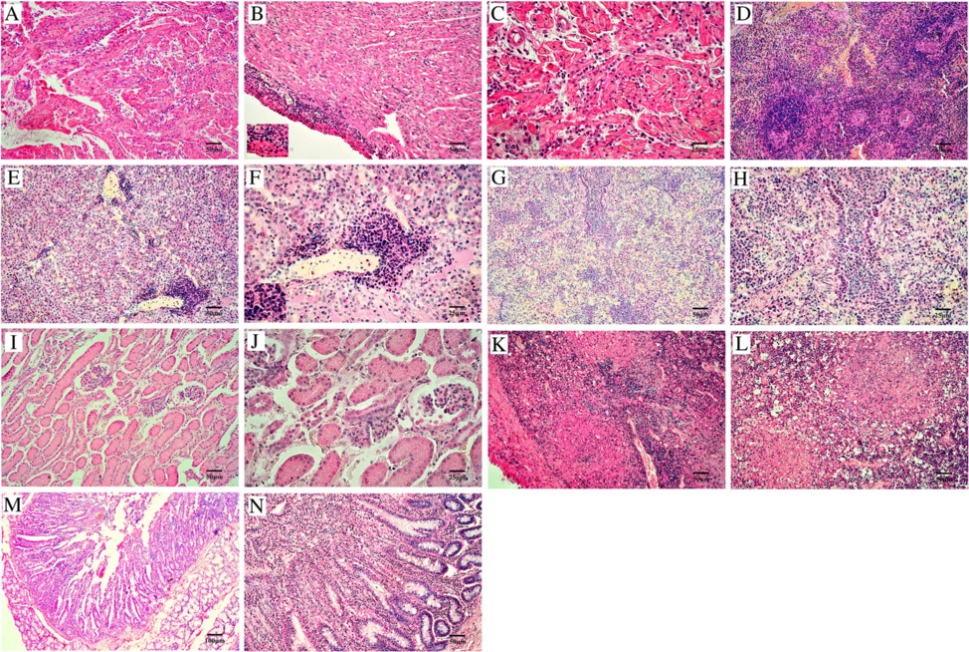
**Histological lesions in multiple organs.** Pathological changes were characterized by hemorrhage, hyperemia, inflammatory infiltration, and necrosis. **(A, B, C)** Myocarditis. **(D)** Dysplasia of the lymphoid follicles in the spleen. **(E, F)** Liver displaying hepatic necrosis and lymphocyte infiltration. **(G, H)** Lung with extensive lymphocyte infiltration, hemosiderosis, hemorrhage, and shed alveolar epithelial cells within the bronchioles and alveoli. **(I, J)** Necrosis and degeneration in the kidney. **(K, L)** Dysplasia, fibrosis, lack of lymphocytes, and necrosis in a lymph node. **(M, N)** Coagulation, necrosis, and abruption of the intestinal villi.

The pathological changes in various tissues were determined with microscopy. The lesions observed in the lung, liver, heart, kidney, lymph node, spleen and intestinal tract tissues were similar in all the pigs necropsied. The heart lesions were characterized as viral myocarditis (Figure 2A). The epicardium was predominantly infiltrated by lymphocytes, with a small number of neutrophils (Figure 2B). Granular myocardial degeneration, edema, and lymphocyte and neutrophil infiltration in the myocardium were observed (Figure 2C). A hepatic examination revealed features characteristic of hepatitis in a number of liver samples, including congestion, vacuolization, and necrosis, (Figure 2E). Lymphocyte and neutrophil infiltration, particularly in the portal area, was clearly observed (Figure 2F). Examination of the lungs demonstrated large areas of hyperemia, hemorrhage, and lymphocyte and neutrophil infiltration, with very little normal histological structure. The bronchioles contained exfoliated alveolar epithelial cells and pink liquid exudate (Figure 2G,H). Enlargement of the glomerulus and focal lymphocyte infiltration were observed in the kidneys. The renal tubule epithelial cells showed granular degeneration and necrosis, and congestion and hemorrhage were present in the kidneys. The renal tubule epithelial cells shed off from the basilar membrane. The glomerulus contained albuminoid droplets of exudate (Figure 2I,J). The organs of immune system were severely underdeveloped, and malformed splenic white pulp was responsible for the reduced numbers of lymphocytes (Figure 2D). Poorly developed lymph nodes were also evident. The majority of capillaries were expanded and hyperemia was present. The lymphoid nodules were smaller than normal, resulting from fibrosis, necrosis, and lymphocyte depletion (Figure 2K, L). Examination of the intestine revealed necrosis, and coagulation of the intestinal villi. The submucosal layer was exposed due to the loss of mucosal layer. Epithelial cell shedding and secretion from the intestinal glands into the gut cavity were increased (Figure 2M, N). The main pathological changes observed in the various organs of the seven necropsied pigs are summarized in Table 1.

**Table 1:** **Main pathological changes in the organs of the seven pigs necropsied**

**Organ**	**Pathological changes**	**Pig farm A**			**Pig farm B**			
		**1**	**2**	**3**	**4**	**5**	**6**	**7**
		**HEV+**	**HEV+**	**HEV+**	**HEV+**	**HEV+**	**HEV-**	**HEV-**
		**PCV+**	**PCV-**	**PCV+**	**PCV+**	**PCV+**	**PCV+**	**PCV+**
Liver	Degeneration	**+**	**+**	**+**	**+**	**+**	**+**	**+**
	Edema	**+**	**+**	**-**	**+**	**+**	**+**	**-**
	Congestion	**+**	**+**	**+**	**+**	**+**	**+**	**+**
	Hemorrage	**-**	**+**	**-**	**-**	**-**	**+**	**-**
	Necrosis	**+**	**+**	**+**	**+**	**+**	**+**	**+**
	Inflammation	**+**	**+**	**+**	**+**	**+**	**+**	**+**
	Fibrosis	**-**	**-**	**-**	**-**	**-**	**-**	**-**
Heart	Degeneration	**+**	**+**	**+**	**+**	**+**	**+**	**+**
	Edema	**+**	**-**	**+**	**-**	**+**	**+**	**+**
	Necrosis	**+**	**+**	**-**	**-**	**+**	**+**	
	Inflammation	**+**	**+**	**-**	**+**	**-**	**-**	**+**
Lung	Degeneration	**+**	**+**	**+**	**+**	**+**	**+**	**+**
	Congestion	**+**	**+**	**+**	**+**	**+**	**+**	**+**
	Hemorrage	**+**	**+**	**+**	**+**	**+**	**+**	**+**
	Necrosis	**+**	**+**	**+**	**+**	**+**	**+**	**+**
	Inflammation	**+**	**+**	**+**	**+**	**+**	**+**	**+**
	Exfoliation of alveolar epithelial cells	**+**	**+**	**-**	**+**		**+**	**+**
Kidney	Degeneration	**+**	**+**	**+**	**+**	**+**	**+**	**+**
	Edema	**+**	**+**	**+**	**+**	**+**	**+**	**+**

### Pathogen detection

**Figure 3:**
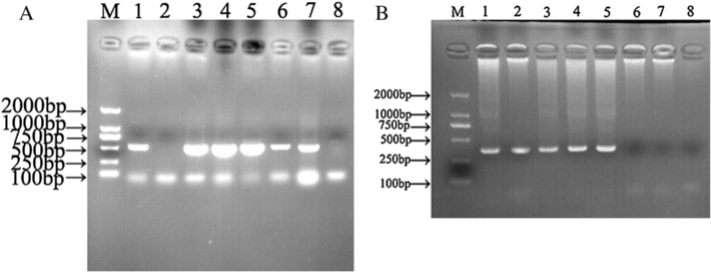
**PCR assays of liver tissues with primers specific for PCV2 and HEV. (A)** PCV2: lane M, DL2000 marker; 1, pig 1 liver; 2, pig 2 liver; 3, pig 3 liver; 4, pig 4 liver; 5, pig 5 liver; 6, pig 6 liver; 7, pig 7 liver; 8, negative control. The PCV2 amplicon was 494 bp. **(B)** HEV: lane M, DL2000 marker; 1, pig 1 liver; 2, pig 2 liver; 3, pig 3 liver; 4, pig 4 liver; 5, pig 5 liver; 6, pig 6 liver; 7, pig 7 liver; 8, negative control. The HEV amplicon was 348 bp.

PCR was used to detect any viruses in the liver, lung, spleen, brain and kidney samples (Table 1). Viral pathogens responsible for suspicious diseases in swine were investigated: HEV, PCV2, *Classical swine fever virus* (CSFV), *Porcine epidemic diarrhea virus* (PEDV), *Transmissible gastroenteritis coronavirus* (TGEV), *Pseudorabies virus* (PRV), *Porcine parvovirus* (PPV), and PRRSV. PCV2 was detected in the livers of six of the seven pigs (GenBank accession nos. KJ534661, KJ534662, KJ534659, KJ534658, KJ534663, and KJ534660) (Figure 3A) and five of the seven pig livers were HEV positive (GenBank accession nos. KJ123761, KM024042, KJ141160, KJ534657, and KJ534656) (Figure 3B).

**Figure 4:**
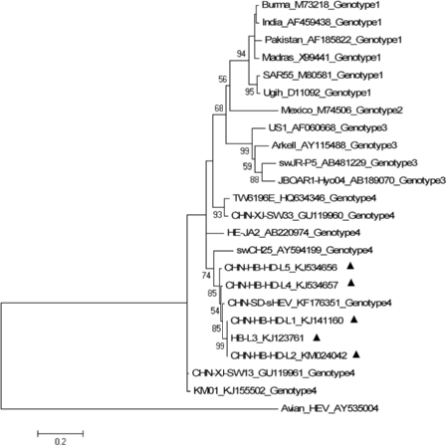
**Phylogenetic analysis based on HEV ORF2 (304 nt, the primers not included) showing the genetic relationships between the isolates identified in this study and isolates from across China and other countries.** A neighbor-joining tree was constructed with bootstrap values calculated from 1,000 replicates. The isolates used for the comparative analysis were HEV genotype 4 strains CHN-SD-sHEV (KF176351), CHN-XJ-SW13 (GU119961), HE-JA2 (AB220974), KM01 (KJ155502), TW6196E (HQ634346), CHN-XJ-SW33 (GU119960), swCH25 (AY594199), genotype 3 strains swJR-P5 (AB481229), Arkell (AY115488), JBOAR1-Hyo04 (AB189070), genotype 2 strain Mexico (M74506), genotype 1 strain India (AF459438), Madras (X99441), Pakistan (AF185822), SAR55 (M80581), Ugih (D11092), US1 (AF060668), Burma (M73218), and Avian HEV (AY535004).

**Figure 5:**
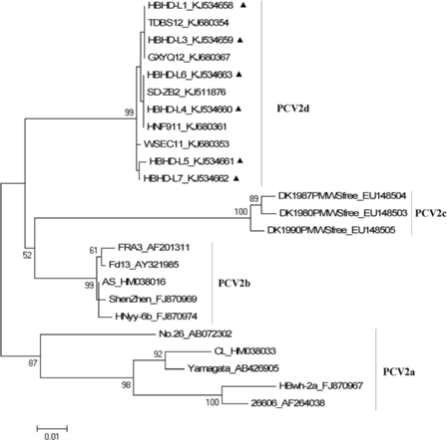
**Phylogenetic analysis based on PCV2 ORF2 (451 nt, the primers not included) showing the genetic relationships between the isolates identified in this study and isolates from across China or other countries.** A neighbor-joining tree was constructed with bootstrap values calculated from 1,000 replicates. The isolates used for the comparative analysis were genotype PCV2d strains HNF911 (KJ680361), SD-ZB2 (KJ511876), WSEC11 (KJ680353), GXYQ12 (KJ680367), TDBS12 (KJ680354), DK1990PMWSfree (EU148505), genotype PCV2c strains DK1987PMWSfree (EU148504), DK1980PMWSfree (EU148503), genotype PCV2b strains AS (HM038016), ShenZhen (FJ870969), HNyy-6b (FJ870974), FRA3 (AF201311), Fd13 (AY321985), genotype PCV2a strains CL (HM038033), HBwh-2a (FJ870967), 26606 (AF264038), Yamagata (AB426905), and No. 26 (AB072302).

A phylogenetic analysis based on the 348-nt open reading frame (ORF) 2 of HEV was used to establish the genetic relatedness of the strains isolated in this study to representative isolates of the four HEV genotypes. Isolates CHN-HB-HD-L1, CHN-HB-HD-L2, HB-L3, CHN-HB-HD-L4, and CHN-HB-HD-L5 were identified as genotype 4, and were most closely related to swCH25 (AY594199), CHN-SD-sHEV (KF176351), HE-JA2 (AB220974) (Figure 4). A phylogenetic analysis based on the 494-nt ORF2 of PCV2 was used to establish the genetic relatedness of the strains isolated in this study to PCV2 strains isolated globally, including in China. The HBHD-L1, HBHD-L3, HBHD-L4, HBHD-L5, HBHD-L6, and HBHD-L7 (Figure 5) isolates were closely related to genotype PCV2d strains HNF911 (KJ680361), SD-ZB2 (KJ511876), WSEC11 (KJ680353), GXYQ12 (KJ680367), TDBS12 (KJ680354). Other tissues were also tested using PCR. The liver, spleen, kidney, lung, and brain of pig No. 1, 2, 3, 4, and 5 were positive for HEV RNA and these tissues were PCV2 DNA positive in pig No. 1, 3, 4, 5, 6, and 7. The co-infection rate for HEV and PCV2 was 57.1% (4/7). The livers, lungs, kidneys, and spleens of the necropsied pigs were negative for PEDV, TGEV, CSFV, PRRSV, PRV, and PPV.

### Immunohistochemistry

**Figure 6:**
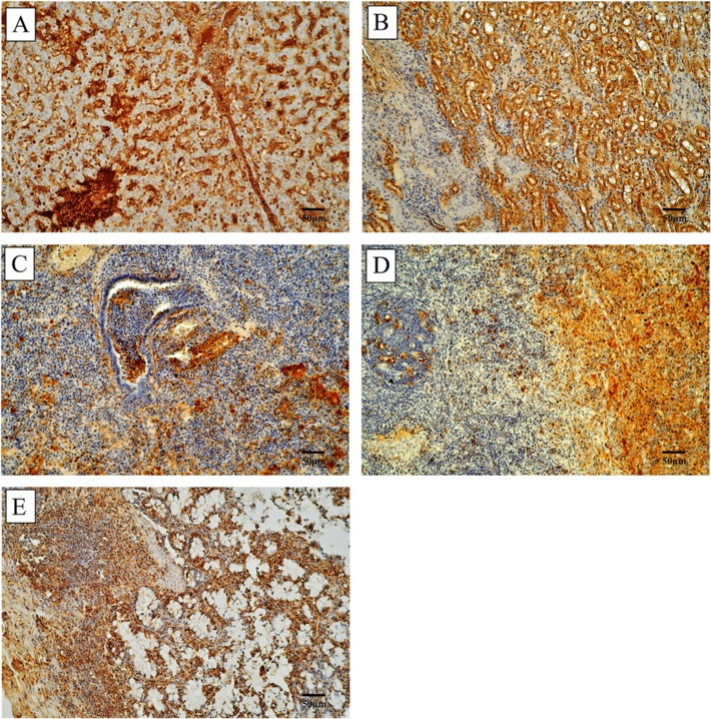
**Detection of HEV antigen in different organs of the dead swine. (A)** Positive HEV signal in the liver. **(B)** Positive HEV signal in the kidney. **(C)** Positive HEV signal in the lung. **(D)** Positive HEV signal in a lymph node. **(E)** Positive HEV signal in the intestine.

**Figure 7:**
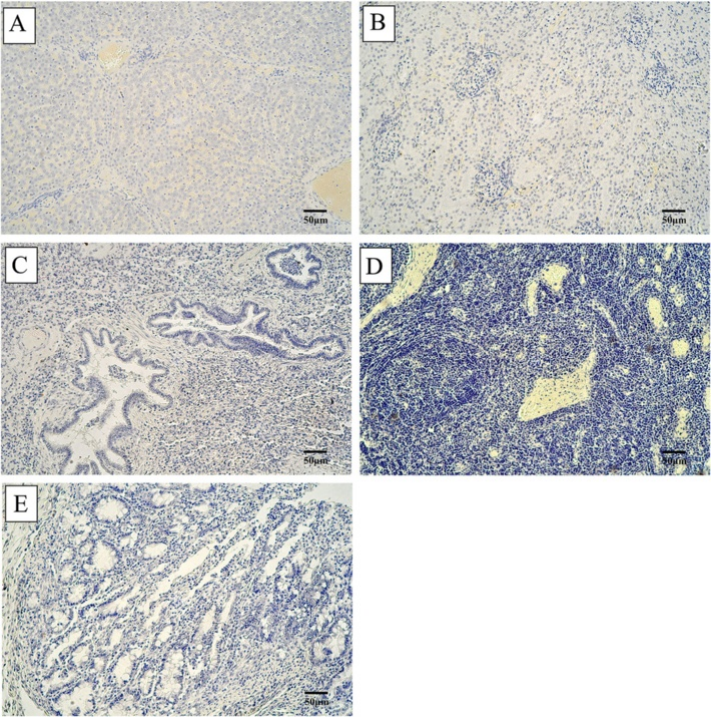
**Negative controls for IHC analysis of different tissues. (A)** Liver. **(B)** Kidney. **(C)** Lung. **(D)** Lymph node. **(E)** Intestine.

Immunohistochemical (IHC) staining confirmed the presence of HEV and PCV2 antigens in several tissues and organs. HEV antigen was detected in the livers, kidneys, lung, intestine and lymph nodes of all five HEV-positive swine (pig No.1, 2, 3, 4, 5). Granular or diffuse positive staining was seen in the hepatic sinusoid and the cytoplasm of hepatocytes (Figure 6A). The nuclei and cytoplasm of the renal tubular epithelial cells (Figure 6B) and lung cells (Figure 6C) were positive for HEV antigen. The staining for HEV antigen in the lymph nodes was intense in the lymphocytes and macrophages (Figure 6D). The staining for HEV antigen in the intestinal tissue was intense in the lamina propria and gut-associated lymphoid tissue (Figure 6E). The negative control is shown in Figure 7. HEV antigen was negative in the two HEV RNA negative swine (pig No.6 and 7).

**Figure 8:**
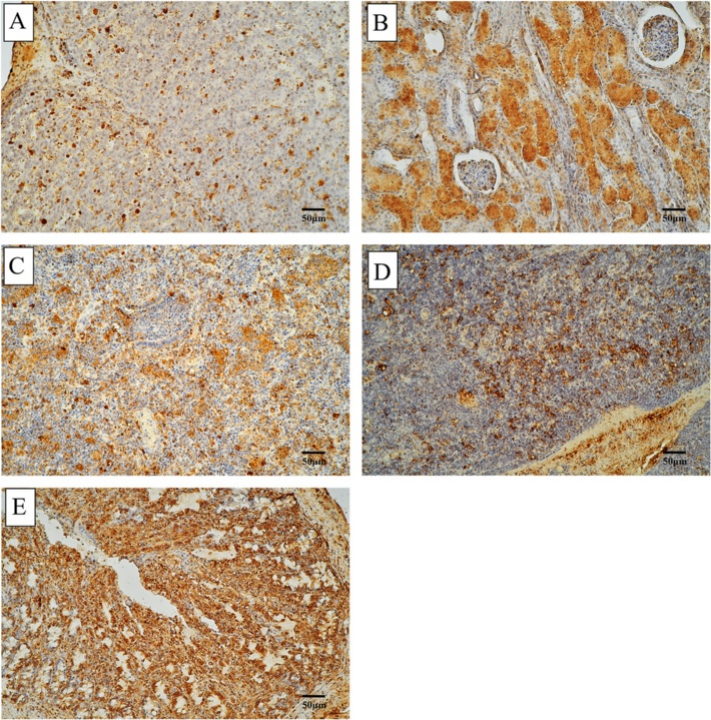
**Detection of PCV2 antigen in different organs of the dead swine. (A)** Positive PCV2 signal in the liver. **(B)** Positive PCV2 signal in the kidney. **(C)** Positive PCV2 signal in the lung. **(D)** Positive PCV2 signal in a lymph node. **(E)** Positive PCV2 signal in the intestine.

The lungs, livers, kidneys, lymph nodes, and intestine were tested for PCV2 antigen with IHC staining. The tissue distribution of the PCV2 antigen was similar in all PCV2 DNA positive pigs (pig No.1, 3, 4, 5, 6, 7). In the liver, PCV2 antigen was detected within the hepatocytes and Küpffer cells (Figure 8A); in the kidneys, the positive signals were in the tubular epithelial cells (Figure 8B); and for the lungs, PCV2-antigen positive signals were in the alveolar and septal macrophages, and fibroblast-like cells in the lamina propria of the airways (Figure 8C). PCV2 antigen was intense in the lymphocytes and macrophages in the lymph nodes (Figure 8D), and the mucous layer and lamina propria of the intestine (Figure 8E). The negative control is shown in Figure 7. PCV2 antigen was negative in the PCV2 DNA negative pig (pig No.2).

## Conclusions

*Hepatitis E virus* infections are a major cause of acute hepatitis in developing countries, and because of the zoonotic transmission of HEV, they are also an emerging health problem in industrialized countries. Swine are considered to be a major reservoir of the HEV transmitted to humans [3,11]. Four main genotypes have been identified in HEV. Genotypes 1 and 2 have only been found in humans, whereas genotypes 3 and 4 have been recovered from both humans and pigs [12]. Smith et al. recently proposed a taxonomic scheme, which divided the family Hepeviridae into the genera Orthohepevirus (all mammalian and avian hepatitis E virus (HEV) isolates) and Piscihepevirus (cutthroat trout virus) [13]. The livers of pigs naturally infected or intravenously inoculated with HEV display focal lymphocytic infiltration and swollen, vacuolated hepatocytes [14]. The livers of the seven pigs investigated in the present study had significant lymphocytic infiltration in the portal area, and large localized areas of fibrosis, necrosis, and vacuolization. IHC staining showed that the ORF2 protein of HEV was distributed across multiple organs, particularly in the liver and kidneys. This result was not unexpected because the liver is the target organ of HEV, and the kidney plays an integral role in maintaining extracellular fluid homeostasis. The previous study also demonstrates that HEV has been found in liver and kidney after experimental infection in domestic pigs [15]. A PCR assay specific for HEV ORF2 confirmed that the pigs were HEV positive. Isolates CHN-HB-HD-L1, CHN-HB-HD-L2, HB-L3, CHN-HB-HD-L4, and CHN-HB-HD-L5 were shown to belong to genotype 4, the most prevalent HEV genotype in China. According to Smith et al. [13], the HEV strains isolated in our case were classified to Orthohepevirus A.

PCV2 is the primary causative agent of PMWS which was first described in Canada in 1991 [16]. In recent years, PMWS has become a serious economic problem for the swine industry in China. According to the data from a prevalence survey, more than 67.1% of piglet stool samples were PCV2 positive [17]. The disease predominantly affects pigs between 5 and 15 weeks of age and is characterized by growth retardation, diarrhea, dyspnea, jaundice, and enlargement of the inguinal lymph nodes. In our study, infected swine aged 2–3 months displayed clinical symptoms consistent with previous reports of the disease [9].

In this study, the clinical and pathological changes observed were consistent with typical PCV2 infection. Hemorrhage, hyperemia, edema, necrosis, and lymphocyte infiltration were observed in all organs, most notably the lungs. Histological changes consistent with lobar pneumonia were also evident in the lungs, and normal lung histology was rarely seen. The alveolar walls were thickened, with substantial lymphocyte, erythrocyte, and exudate infiltration. Exfoliated alveolar epithelial cells and pink liquid exudate were observed within the bronchioles. PCV2 has a small, nonenveloped icosahedral virion, and a single-stranded circular DNA genome, 1,767–1,768 nt in length. The genome has two major ORFs encoded in the antisense direction [18]. Isolates HBHD-L1, HBHD-L3, HBHD-L4, HBHD-L5, HBHD-L6, and HBHD-L7 recovered in this study were closely related to the genotype PCV2d strains HNF911 (KJ680361), SD-ZB2 (KJ511876), WSEC11 (KJ680353), GXYQ12 (KJ680367), TDBS12 (KJ680354) (Figure 4). The genotype PCV2d represented a novel genotype and a shift from PCV2a to PCV2b as the predominant genotype in China in recent years [19]. A genetic analysis, combined with the observed pathological changes, indicated that the PCV2 isolates detected in this study were probably high prevalent in China [20]. IHC staining of tissues for the ORF2 protein of PCV2 revealed that the antigen was observed in the lungs, liver, lymph node, intestine and kidneys, further evidence of PCV2 infection.

Further pathological changes typical of PCV2 infection were observed in this study. Significant immune-system-organ dysplasia was apparent, with the characteristic histopathological findings of lymphoid depletion and histiocytic replacement in the lymphoid tissues. Combined with the positive PCV2 ORF2 signals in lymph node in IHC, these results suggest that the systemic immune function of these pigs had been disrupted.

IHC staining for HEV and PCV2 antigens revealed a diffuse labeling pattern in the intestine, with the greatest reactivity observed in the cytoplasm of cells in the mucous layer and lamina propria. This observation is consistent with the viral invasion pathways. The transmission of HEV occurs via the fecal–oral route, so HEV may invade the animal through the intestinal mucous layer, with infection progressing to the lamina propria. We have investigated the mucosal immunity in the intestines of rabbits [21] and gerbils (data not shown) experimentally infected with HEV, and both studies demonstrated a strong HEV ORF2 positive signals in intestinal. In the present study, naturally infected swine exhibited significant necrosis of the intestinal epithelial cells and also showed HEV ORF2 positive signals in intestine. Therefore, HEV invasion of the intestine may proceed rapidly and widely, consistent with the diffuse labeling pattern observed in the intestine in this study.

We also tested for other suspicious pathogens in this study. According to the medical history, the sick piglets failed to respond to antibiotic treatment (timicosin and doxycycline in pig farm A, and florfenicol in pig farm B), indicating that bacterial infection was unlikely. PRRSV, CSFV, PEDV, TGEV, PRV, and PPV are high prevalent in swine in China and across the globe. Although infections with these viruses may present with similar clinical symptoms, including fever, diarrhea, depressed, and decrease of feed intake. PCR confirmed that all seven pigs were negative for these viruses.

Presence of signs and lesions such as lymphoid depletions, hepatitis, nephritis, etc. resemble the microscopic characteristic of PMWS. Nevertheless, no typical microscopic lesions such as granulomatous inflammation or intracytoplasmic inclusion bodies were observed in lymphoid tissue, liver, spleen, and other tissues [22]. The mortality associated with PCV2 infection is generally around 10% (range 4%–20%), but can reach 50% [23]. In our case, seven pigs were detected and the pathogens had been identified. However, the true reason for the high mortality in the whole pig farm A and B still need more exploring. The occurrence of this disease may not be only a matter of PMWS caused by PCV2 infection. The previous study showed swine HEV infection can be a significant factor to the development of hepatitis regardless of the PMWS status [8]. In addition, J. Ellis [24] claimed that the severity of hepatic lesions in PCV-2 infected pigs may be enhanced by co-infection with swine hepatitis E virus. It may indicate that the significance of HEV is hardly negligent. Further investigation about the mechanistic basis for the pathogenesis of the clinical syndrome that associated with PCV2 and HEV co-infection needs to be conducted.

HEV infection in humans and animals is common, but the natural occurrence of HEV and PCV2 co-infection in pigs in China, reported here, has rarely been seen. The experimental infection of domestic pigs with HEV did not cause death [25]. Therefore, the HEV-infected swine observed in this study requires further investigation. Based on these results, we believe that considerable attention should be directed towards co-infections of HEV and PCV2 in swine.

According to the comparison of histopathological changes between these cases in Table 1, no specific characteristics are demonstrated, which is really thought-provoking. It is very significant to explore the reasons for the similar pathological changes in the HEV and/or PCV2 infected pigs. In order to reveal the mechanism for the similar pathological changes in the HEV and/or PCV2 infected pigs, further tests about HEV and PCV2 co-infection, single HEV infection and single PCV2 infection in pigs have been in the planning. We hope to reveal the mechanism of the similar pathological changes and also discover the similarity and differences between the natural cases and the experimental infected pigs. To our best knowledge, theses following reasons are speculated to explain the similar pathological changes: a) the individual differences in pigs. Different pigs may have different reaction to the attack of the viruses; b) pig No.2 may once have been infected with PCV2 and then PCV2 were neutralized by the antibody. But lesions in the tissues hadn’t recovered when it died; c) HEV is RNA virus without envelope. It may have degraded in tissues in pig No.6 and 7 before testing. d) the complicate in natural infected cases.

In conclusion, co-infection of HEV and PCV2 were identified in four out of seven dead weaned pigs from two pig farms in China. Severe pathological changes and high mortality were observed in the infected animals. Our results indicate that co-infection with HEV and PCV2 may bring some negative effect on the swine industry in China, and this phenomenon requires further investigation. What’s more, further research is required to demonstrate the role of co-infection of HEV and PCV2 in swine and whether these two viruses exert a synergistic effect.

## Materials and methods

### Consent

Written informed consent was obtained from the owners of the two farms for the publication of this report and any accompanying images.

### Determination of pathogen

According to the gross and histopathological lesions, eight suspected viruses were detected. Total RNA and DNA were extracted from liver, lung, kidney, brain, and spleen specimens using the UltraPure™ RNA Kit and the General AllGen Kit (CWBIO, Beijing, China), according to the manufacturers’ instructions. The extracted RNA was used in reverse-transcription polymerase chain reaction (PCR) assays to detect HEV, PRRSV, PEDV, TGEV, and CSFV. The extracted DNA was used to detect PCV2, PRV, and PPV. The virus-specific primers used in this study are listed in Table 2. PCR for PRRSV, PEDV, TGEV, CSFV, PCV2, PRV, and PPV, included initial denaturation at 94°C for 5 min, followed by 35 cycles of 94°C for 30 sec, 55°C for 30 sec, and 72°C for 30 sec, with a final extension step at 72°C for 10 min. PCR for HEV included initial denaturation at 95°C for 7 min, followed by 35 cycles of 94°C for 1 min, 42°C for 1 min, and 72°C for 2 min, with a final extension step at 72°C for 10 min. Sterile ddH_2_O (1 μL) was included as the negative control.

**Table 2:** **Sequences of primers used in the PCR assays**

**Primer**	**Sequence 5’-3’**	**Reference**
PRRSV ORF7 gene		[26]
PRRSV-F	CCAAATAACAACGGCAAGCA	
PRRSV-R	ATGCTGAGGGTGATGCTGTGA	
PCV2 ORF2 gene		[27]
PCV2.S4	CACGGATATTGTAGTCCTGGT	
PCV2.A4	CCGCACCTTCGGATATACTGTC	
HEV ORF2 gene		[3]
HEV- externer primer	AATTATGCYCAGTAYCGRGTTG	
HEV- externer primer	CCCTTRTCYTGCTGMGCATTCTC	
HEV- interner primer	GTWATGCTYTGCATWCATGGCT	
HEV- interner primer	AGCCGACGAAATCAATTCTGTC	
CSFV E2 gene		[28]
E2- externer primer	GCATCAACCAYKGCATTCC	
E2- externer primer	GTCTGTGTGGGTRATTAAGTTCCCTA	
E2- interner primer	CTRGTRACTGGGGCACAAGG	
E2- interner primer	ACCAGCRGCGAGTTGYTCTG	
PEDV S gene		[29]
P1	TTCTGAGTCACGAACAGCCA	
P2	CATATGCAGCCTGCTCTGAA	
TGEV S gene		[29]
T1	GTGGTTTTGGTYRTAAATGC	
T2	CACTAACCAACGTGGARCTA	
PRV gB genes		[30]
PRVF	GGGGTTGGACAGGAAGGACACCA	
PRVR	AACCAGCTGCACGCGCTCAA	
PPV NS1 gene		[30]
PPVF	AGTTAGAATAGGATGCGAGGAA	
PPVR	AGAGTCTGTTGGTGTATTTATTGG	

### Sequencing and phylogenetic analysis

The PCR products were purified and ligated into the pMD18-T vector (TaKaRa, Dalian, China), and sequenced on an ABI PRISM® 377 DNA Sequencer using reagents from BGI Life Technologies (Beijing, China). The sequences were analyzed with DNAMAN (version 5.2.2; Lynnon Corp., Quebec, Canada). Phylogenetic analyses were performed with the MEGA software (version 4.0; http://www.megasoftware.net) using the neighbor-joining method with 1000 bootstrap replicates.

### Immunohistochemistry

Immunohistochemistry was used to detect HEV and PCV2 antigens in the liver, kidney, lymph nodes, intestine and lung. The endogenous enzymatic activity in the tissues was blocked with 5% H_2_O_2_. Goat serum was used to block the Fc receptors in the tissues. The primary antibody, monoclonal mouse anti-HEV ORF2 antibody (1:300 dilution; Beijing Protein Institute, Beijing, China), was added to the sections and incubated at 37°C for 2 h. IHC staining was performed with the Histostain™-Plus Kit (ZSGB-BIO, Beijing, China), according to the manufacturer’s instructions. The substrate 3,3’-diaminobenzidine tetrahydrochloride (ZSGB-BIO) was applied for 10 min, after which Gill’s hematoxylin counterstain was added. A monoclonal mouse anti-PCV2 ORF2 antibody (1:200 dilution; kindly supplied by Dr. Liu Jue, Beijing Municipal Key Laboratory for the Prevention and Control of Infectious Diseases in Livestock and Poultry, Beijing, China) was used to detect PCV2. For the negative controls, the primary antibody was omitted and replaced with phosphate-buffered saline. In another negative control, the primary antibody was replaced with IgG from a normal mouse to demonstrate the specificity of the signal.

## Abbreviations

HEV: *Hepatitis E virus*
HE: Hepatitis E
PCV2: Porcine circovirus 2
PRRSV: Porcine reproductive and respiratory syndrome virus
CSFV: Classical swine fever virus
PEDV: Porcine epidemic diarrhea virus
TGEV: Transmissible gastroenteritis coronavirus
PRV: *Pseudorabies virus*
PPV: *Porcine parvovirus*
PMWS: Postweaning multisystemic wasting syndrome
